# Comparative In Vitro Evaluation of Anti-HIV Immunotoxin, Antibody–Drug Conjugate, and Radioimmunoconjugate Targeted by the Same Antibody

**DOI:** 10.3390/antib15010012

**Published:** 2026-01-28

**Authors:** Anne-Sophie Kuhlmann, Tami Peters, Donald K. Hamlin, Yawen Li, Xinyi Wang, Megan Stackhouse, Frances M. Cole, Jasmin Martinez-Reyes, Brenda M. Sandmaier, Hans-Peter Kiem, D. Scott Wilbur, Robert D. Harrington, Seth H. Pincus

**Affiliations:** 1 Translational Science and Therapeutics Division, Fred Hutchinson Cancer Center, Seattle, WA 98109, USAbsandmai@fredhutch.org (B.M.S.); hkiem@fredhutch.org (H.-P.K.); 2 Department of Chemistry & Biochemistry, Montana State University, Bozeman, MT 59717, USA; 3 Department of Radiation Oncology, School of Medicine, University of Washington, Seattle, WA 98195, USA; dkhamlin@uw.edu (D.K.H.); liyw@uw.edu (Y.L.); dswilbur@uw.edu (D.S.W.); 4 Department of Medicine, University of Washington School of Medicine, Seattle, WA 98195, USA; rdh@uw.edu

**Keywords:** HIV reservoir eradication, monoclonal antibody, cytotoxic immunoconjugate, antibody-drug conjugate, immunotoxin, radioimmunoconjugate, immunotherapy, metabolomics, RNAseq, HIV envelope protein, gp160, gp41

## Abstract

Background: We are developing cytotoxic immunoconjugates (CICs) to eliminate HIV-infected cells. We investigated the efficacy and kinetics of killing by different forms of CICs targeted by the same monoclonal antibody (mAb), an immunotoxin (IT), antibody-drug conjugate (ADC), and radioimmunoconjugate (RIC). Methods: We compared in vitro effects of CICs made by conjugating anti-gp41 mAb 7B2 to deglycosylated ricin A chain (7B2-dgA), the anthracycline derivative PNU-159682 (7B2-PNU), or the α-emitting isotope actinium-225 (7B2-^225^Ac). Kinetic analyses of cell growth were performed measuring electrical impedance every 15 min over a 7-day period using cells stably expressing the HIV envelope and Env-negative parent cells. Results: 7B2-dgA and 7B2-^225^Ac were more potent and acted more rapidly to kill cells than 7B2-PNU. Both the 7B2-PNU and 7B2-^225^Ac induced bystander-cell killing, whereas the IT did not and consequently allowed the outgrowth of Env-negative cells. Low dose or brief exposure to 7B2-PNU resulted in an increased rate of cell growth. Conclusions: An IT, ADC, and RIC showed substantial differences in the degree of specific toxicity, kinetics, and mechanisms of killing. The results of this side-by-side comparison have implications for the development of CICs to treat HIV, as well as other conditions.

## 1. Introduction

Cytotoxic immunoconjugates (CICs) are bifunctional molecules consisting of a targeting domain, often a monoclonal antibody (mAb), and a toxic moiety. The function of therapeutic CICs with tropism defined by the antibody moiety is to target and lyse specific cells such as cancer cells, while sparing healthy cells. Depending upon the toxic moiety used, these CICs are termed differently: an immunotoxin (IT) when a protein toxin of plant or bacterial origin is used, an antibody–drug conjugate (ADC) in the case of a small cytotoxic molecules, or a radioimmunoconjugate (RIC) if a high-energy nuclide is bound to the mAb. To function effectively, ITs and ADCs must be internalized to allow the toxic moiety to reach its intracellular target; RICs do not [1]. Initial studies of CICs were performed using ITs, which proved to be highly immunogenic and therefore of limited clinical utility [2,3,4]. While the use of ADCs or RICs avoids this problem to a large degree, the question remains whether ITs might still offer therapeutic advantages if optimized to decrease their immunogenicity. To this end, efforts have been made to de-immunize toxins through the elimination of B- and T-cell epitopes, or by using pharmacological means to suppress the anti-CIC immune response [3,5,6].

CICs have been successfully used in the treatment of cancer, immunosuppression, and persistent infections [7,8,9]. We and others have pursued the use of CICs to eliminate HIV-infected cells that persist despite years of fully suppressive anti-retroviral therapy (ART) [4,10,11,12,13,14,15,16,17,18]. This reservoir, which renews HIV replication and viremia soon after the cessation of ART, represents a major barrier to curing HIV infection. If activated, these infected cells express only a single viral protein on their surface: the envelope protein (Env or gp160) consisting of an extracellular domain, gp120, and a transmembrane subunit gp41. Anti-HIV monoclonal antibodies (mAb) have been proposed and tested in strategies to deplete the reservoir of HIV infection that persists despite effective antiretroviral therapy [12,15,19,20,21,22,23,24,25,26,27]. Cytotoxic mAbs kill cells by Fc-mediated complement fixation, antibody-dependent cellular cytotoxicity (ADCC), or antibody-dependent phagocytosis. However, not all anti-HIV mAbs elicit Fc-mediated effects, or they do so incompletely due to impaired cellular immune responses. As an alternative approach, we set out to develop CICs with enhanced cytotoxicity compared to unmodified mAbs. We screened >200 mAbs directed against the HIV-1 Envelope (Env) and identified the external helix–loop–helix region of gp41 as an optimal target for CICs [11].

We and others have tested ITs, showing cytotoxic and antiviral activity in vitro at clinically obtainable concentrations, and therapeutic effects in humanized murine models of HIV infection [4,18,28,29]. We have also shown efficacy in SHIV-infected macaques, but the effect was blunted by immunogenicity [4]. We have subsequently used several approaches to address the immunogenicity of ITs, including the development of ADCs and RICs. An important difference in developing ADCs directed against HIV versus those targeting malignant cells is that cells actively producing HIV or expressing HIV Env need not be proliferating, while malignant cells are, by definition, dividing and therefore susceptible to toxins or drugs that disrupt cell replication. Our initial attempts to produce anti-HIV ADCs resulted in agents that required high drug concentrations for cytotoxic efficacy [4]. More recently, we identified the anthracycline derivative PNU-159682 (PNU) as a drug that kills resting lymphocytes and used it to produce an effective ADC targeting Env-expressing cells. We also showed that the ADC and an identically targeted IT differed in the kinetics of killing, with the ADC requiring more time to kill cells [30]. RICs are particularly effective reagents since they do not require internalization for cell killing, they can effectively kill resting cells, and they are less immunogenic than toxin- or drug-based immunoconjugates [29,31,32]. We conjugated the anti-HIV Env mAb 7B2 to alpha-emitting radionuclides with different half-lives and identified 7B2-^225^Actinium (7B2-^225^Ac) as an effective RIC [33]. We propose that these CICs could be used in combination with latency disrupting agents that would activate persistently infected cells to secrete virus and express Env, thereby becoming targets of the CICs in an “activate and purge” protocol.

In this investigation, we compared the in vitro cytotoxicity of ADCs, ITs, and RICs made from the anti-gp41 mAb 7B2. We first compared the effects of two different CICs, a well-characterized IT consisting of 7B2 conjugated to a de-glycosylated ricin A chain (7B2-dgA) [12,34], and an ADC consisting of 7B2-PNU [30]. When we developed the effective alpha-emitting RIC, 7B2-^225^Ac, it was added to the comparisons. The high ionizing density of α-particles induces double-stranded DNA breaks that initiate a chain of events causing cell death; therefore, cytotoxicity is independent of cell cycle and dose rate [35]. The effects of these CICs were tested in target cells transfected to constitutively express the cell-surface Envelope of HIV-1 (Env) [11,12]. We studied the kinetics of cell killing and the bystander-cell killing effects of exposure to each CIC. Differences were observed, which could affect the choice of CIC used for further clinical development.

## 2. Materials and Methods

### 2.1. Cell Lines and Antibodies

HEK-293T/92UG cells (hereafter 92UG) constitutively express Env of the Clade A clinical HIV isolate 92UG037.8 in its native conformation. The cells were the gift of Dr. Bing Chen, Boston Children’s Hospital [36]. The expression of Env on the surface of these cells was maintained by periodic selection with puromycin (1 µg/mL). Cells were cultured at 37° in a humidified CO_2_ atmosphere and maintained by serial passage so as not to exceed a concentration of 10 × 10^6^ cells/mL. HEK-293T ((ATCC, VA, USA) CRL-3216) and 92UG cells were grown in DMEM (Gibco Thermo Fisher, Waltham, MA, USA, 4.5 gm glucose/L) with 10% fetal bovine serum (Hyclone, Logan, UT, USA), and 1% penicillin/streptomycin (GIBCO).

MAb 7B2 binds CSGKLIC, the conserved 7 amino acid loop within the helix–loop–helix region of gp41 [37]. 7B2 was originally produced by Dr. James Robinson (Tulane University), and purified 7B2 was the gift of Dr. Barton Haynes (Duke University). 7B2-dgA consisted of deglycosylated ricin A chain (dgA), the kind gift of Ellen Vitetta (University of Texas Southwestern), conjugated to 7B2 using the heterobifunctional cross-linking agent succinimidyl 6-[3(2-pyridyldithio) propionamido] hexanoate (SPDP, Pierce Biotechnology, Rockford, IL, USA) at a 1:1 mass ratio, as described previously [4]. Deglycosylating ricin A chain avoids the non-specific toxicity of the A chain-associated glycan that binds mannan receptors on hepatocytes [38]. Products were characterized biochemically by SDS-PAGE and contained a mixture of 0, 1, 2, and 3 dgA per 7B2. 7B2-PNU was produced at Levena Biopharma (CA, USA), as described elsewhere [30]. The preparation contained a mixture of 0, 1, 2, and 3 PNUs per 7B2, similar to that of 7B2-dgA. It should be noted that the linker in 7B2-dgA contains a disulfide bond, which may be cleaved, whereas the linker in 7B2-PNU is non-cleavable. 7B2-Py4Pa, a non-cytotoxic immunoconjugate made in preparation for use the construction of 7B2-^225^Ac, was prepared by the conjugation of 7B2 with an 8-fold molar equivalent of H4py4pa-NCS [39]. Conjugates were extensively demetallated by dialysis. Purity of the conjugates was assessed by size exclusion–HPLC. ^225^Ac (t1/2 = 9.92 d) was obtained from the DOE Isotope Program’s National Isotope Development Center as a solid nitrate salt and was used as obtained. Demetallated HEPES buffer (0.05 M, pH 7) was combined with 7B2-Py4pa. To this, 25–35 µL of ^225^Ac (212–430 µCi) in 0.1 M HNO3 was added. The mixture was incubated for 1 h at 37 °C. Labeled mAb was separated from unbound ^225^Ac on a PD-10 column and collected in PBS. Thin-layer chromatography was used to determine the proportion of label incorporated into the RIC (>90%). The amount of radioactivity in the final samples of RIC was quantified by gamma spectroscopy. Final labeling efficiency indicated one ^225^Ac atom per 82 antibody molecules. Full details of RIC conjugation procedures and reagents are described elsewhere [33].

### 2.2. Cell-Surface HIV Envelope Expression by Flow Cytometry

Following trypsinization, 2 × 10^5^ cells from each cell line were taken for flow cytometry surface-staining of the HIV envelope. Cells were incubated with 1 mL of 20% FBS-PBS for 10 min at 4 degrees to block the FcR. Cells were washed once with 1 mL 1X PBS and incubated with 2 µg/100 μL of an HIV envelope binding mAb (Anti-HIV-1 gp120 mAb VRC01, NIH AIDS Reagent Program #12033) [40] for 25 min at room temperature. Following a second PBS wash, cells were incubated with 2 µg/100 μL of secondary PE Goat anti-Human IgG Fc antibody (Thermo Fisher, Waltham, MA, USA 12-4998-82) for 25 min at room temperature. Cells were washed a final time with PBS, pelleted and run on a BD Celesta or Fortessa flow cytometer, and analyzed using FlowJo 10.10.0 software.

### 2.3. Cellular Cytotoxicity Assay

We measured cell viability in two ways: MTS dye reduction assay, a measure of oxidative phosphorylation; and by cellular impedance using the xCELLigence Real-Time Cell Analysis (RTCA software Pro 2.6.0, Agilent Technologies, Santa Clara, CA, USA). MTS dye reduction was performed on triplicate cultures with 2 × 10^4^ 92UG or HEK 293T (hereafter 293T) cells suspended in 0.2 mL RPMI 1640 (RPMI medium provides a lower non-specific background A_490_ than DMEM) + 10% fetal bovine serum (FBS) in the presence of the CIC and 500 ng/mL of CD4-IgG2 [41], a gift from Paul Madden (Progenics Inc., New York, NY, USA). We have previously shown that soluble CD4, and its mimetics, markedly enhance the cytotoxicity of 7B2-dgA without inducing cytotoxicity alone [11,12,42,43]. Cells were incubated for the time indicated in each experiment. During the final 3 hrs of incubation, MTS/PMS substrate (CellTiter AQueous, Promega, Madison, WI, USA) was added. We used a microplate reader (BioTek, Winooski, VT, USA) to read Absorbance (A) at 490 nm. The formula below was used to calculate percent cytotoxicity:

{1 − [(A_drug_ − A_no cells_)/(A_no drug_ − A_no cells_)]} × 100.

Cellular toxicity was also measured using an xCELLigence Real Time Cell Analysis system (RTCA). Cell lines were treated with Triton-100, unconjugated 7B2, or the CICs 7B2-dgA, 7B2-PNU, or 7B2-^225^Ac at the indicated concentrations. Soluble CD4-IgG2 (500 ng/mL) was present in the CIC-containing cultures unless specifically indicated otherwise. Each condition was performed in triplicate. 293T cells were plated at 2 × 10^4^ cells/well and 92UG cells at 1 × 10^4^ cells/well in 150 μL of culture volume 20–24 h before adding the immunoconjugates of interest in E-plate 16 PET (Agilent Technologies, Santa Clara, CA, USA). Pre-dilutions of the antibodies, immunoconjugates, CD4-IgG2, or triton X-100 were freshly prepared in cell culture media. A total of 50 μL of the pre-dilution was added to each well already containing 150 μL of cell culture. Unless indicated otherwise, 7B2-dgA and 7B2-PNU were present for the full duration of the culture, whereas the RIC was washed out after one hour to avoid the non-specific toxicity that would result from the continued presence of the high-energy alpha particles. Ascorbate (25 mg/mL) was added to RICs to mitigate radiation damage to the mAb. The xCelligence RTCA software was set to measure cellular impedance every 15 min over 7 days. At the end of the experiment, the data were exported to Microsoft Excel and analyzed using Prism 10.6.0 software. Statistical analyses were performed by comparing the area under the curve (AUC) during the growth phase of untreated cultures (100 h).

### 2.4. Data Display and Statistics

Unless otherwise stated, all samples were prepared and analyzed in triplicate. Results are shown as mean and standard error of mean (SEM). If no error bars are visible, then the symbol on the graph is larger than the error. xCelligence assays do not show SEM. We color-coded the immunoconjugates as follows: blue for negative control (either unconjugated 7B2 or no treatment), gold for 7B2-dgA, red for 7B2-PNU, and magenta for 7B2-^225^Ac. Calculations were performed and graphs drawn using Microsoft Excel and GraphPad Prism v9 (GraphPad, Boston, MA, USA).

## 3. Results

**Figure 1:**
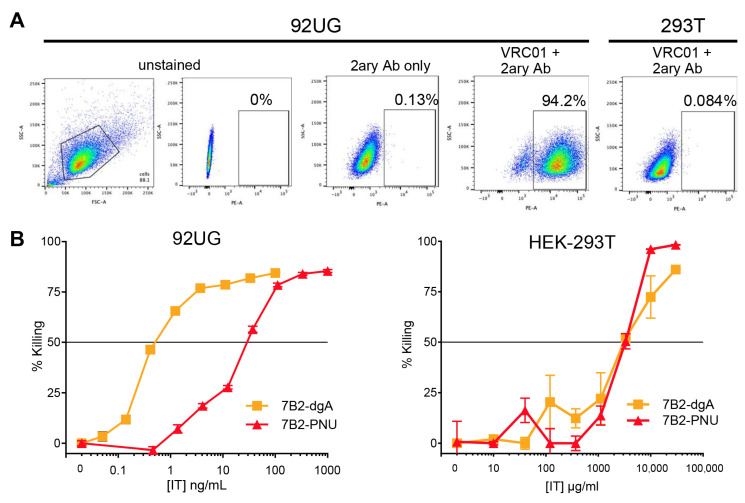
Env expression and cytotoxicity of CICs on 92UG and the parental 293T cells. (**A**) Flow cytometric analysis of cell surface Env. Cells were stained with human anti-gp120 mAb VRC01, then with anti-human IgG secondary Ab labeled with phycoerythrin (PE). The vertical axis shows side scatter on a linear scale. The horizontal axis on the left-most panel is forward scatter on a linear scale. This panel also shows the gate used for analysis of fluorescence. The remaining panels show log fluorescence intensity on the horizontal axis. The percentage of cells within the defined rectangle, i.e., Env+, is indicated. Specificity controls include unstained and only secondary Ab on 92UG cells and fully stained Env− 293T cells. (**B**) Cytotoxicity of CICs. Cells were incubated for three days in the presence of the indicated concentration of CIC and 500 ng/mL CD4-IgG2. Cell viability was quantified as MTS dye reduction over the final 3 h of culture. Note the 100× difference in CIC concentrations tested on 92UG versus 293T. Results show the mean and SEM of triplicate cultures. If no error bars are visible, they are smaller than the symbol.

To compare the cytotoxic effects of 7B2-dgA and 7B2-PNU in cells expressing HIV Env, we evaluated the IT and ADC in 92UG cells, which are 293T cells transfected to constitutively express cell-surface Env in its native conformation [36]. Figure 1A demonstrates the presence of HIV Env on the surface of 94% of the 92UG cells, and the absence of it on the parental HEK-293T cells (or simply 293T), as detected by indirect immunofluorescence using the anti-gp120 mAb VRC01 and flow cytometry. We previously showed that both 7B2-dgA and 7B2-PNU effectively kill persistently infected H9/NL4-3 cells [30]. In Figure 1B, we demonstrate a dose–response of the cytotoxic effects of 7B2-dgA and 7B2-PNU on 92UG and 293T cells. Cytotoxicity was measured as diminished oxidative metabolism, assayed by MTS dye reduction on day 3 of culture. The killing of 92UG cells by 7B2-PNU required roughly 30-fold higher concentrations than that of 7B2-dgA. The non-specific toxicity of both agents on the parental Env-negative cells was equivalent, indicating a ~1000-fold difference between specific and non-specific killing for 7B2-dgA. That ratio was less for 7B2-PNU, but still >100-fold different. This experiment identified the optimal working range of drug concentrations for the kinetics experiments that followed.

**Figure 2:**
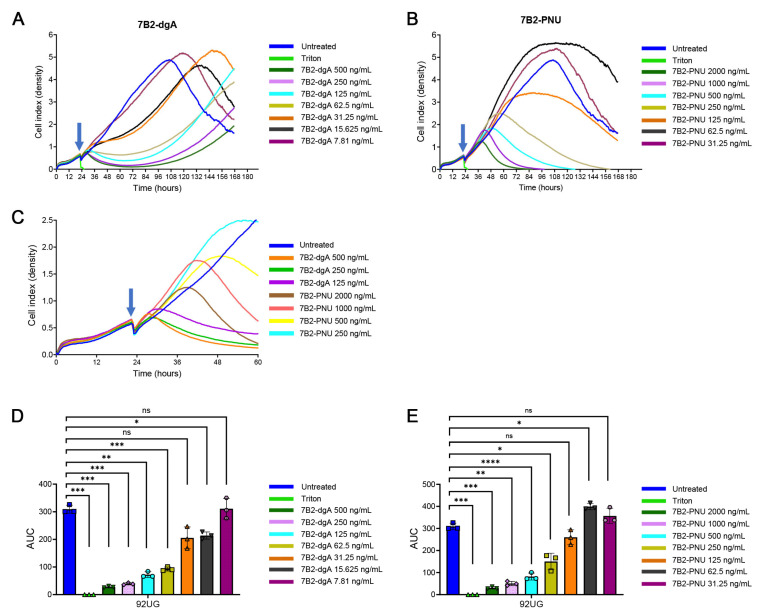
Kinetics of cell killing by immunoconjugates. Ten thousand 92UG cells were plated in triplicate wells. Twenty-four hours later, the indicated CIC plus CD4-IgG2 500 ng/mL was added. Impedance of the monolayer was measured at 15 min intervals and plotted with the *x*-axis indicating time and the *y*-axis used as a measure of monolayer confluence (indicated as a “Cell Density Index”). Each point represents the mean of triplicate wells (no SEM shown). The vertical arrow indicates the time of CIC administration. Panels (**A**,**B**) show the dose–response curves for 7B2-dgA and 7B2-PNU. Triton served as a positive control for cell killing. Panel (**C**) shows the earliest time points of both 7B2-dgA and 7B2-PNU treatment with expanded x and y axes. Panels (**D**,**E**) indicate the area under the curves (AUC) for the dgA (**D**) or PNU (**E**) immunoconjugates between the time of CIC addition and for a duration of 100 h. Two-tailed paired *t*-test statistical analyses were performed using GraphPad Prism. ns: nonsignificant; * *p* < 0.05; ** *p* < 0.01; *** *p* < 0.001; **** *p* < 0.0001.

We next examined the kinetics of cell killing using the xCELLigence RTCA, which measured monolayer density as a function of electrical impedance, assayed every 15 min (Figure 2). A comparison of the dose–response curves for each agent (Figure 2A,B) demonstrates remarkably different patterns. Untreated cells reached peak density at ~100 h and then declined; therefore, statistical differences between curves were calculated over the first 100 h, i.e., the growth period. Cells treated with ≥62.5 ng/mL of 7B2-dgA exhibited growth arrest by 12 h post-treatment, but by 60–70 h there was an outgrowth of cells. An analysis of area under the cell growth curves (AUC) indicates that all concentrations of 7B2-dgA ≥62.5 ng/mL significantly (*p* < 0.01) suppressed growth compared with untreated cells (Figure 2D). In contrast, cells exposed to 7B2-PNU at concentrations ≥125 ng/mL did not attain growth arrest until 24+ hours post-treatment, but then resulted in complete killing with no outgrowth. AUC analyses demonstrated that concentrations above 250 ng/mL mediated significantly different lyses efficiency (*p* < 0.05) from that observed in untreated cells (Figure 2E). Surprisingly, lower concentrations of 7B2-PNU (62.5 and 31.3 ng/mL) significantly enhanced the growth of 92UG cells (*p* < 0.05 at 62.5 ng/mL). The difference between 7B2-dgA and 7B2-PNU in the early time points post-treatment is highlighted in Figure 2C, and confirms our earlier observations that 7B2-dgA killed cells more rapidly than 7B2-PNU [30].

**Figure 3:**
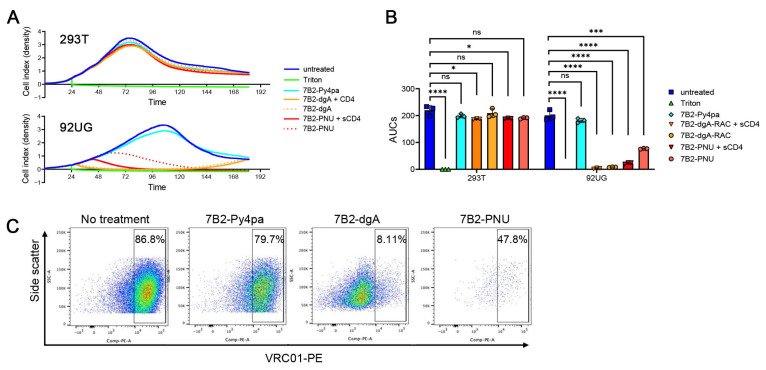
Specificity, CD4 enhancement, and Env expression on cell outgrowth. (**A**) Specificity of killing was demonstrated by absence of effect of the CICs on Env-negative 293T cells and of 7B2-Py4pa (a non-cytotoxic conjugate) on 92UG. Cells were incubated continually in the presence of 7B2-dgA (50 ng/mL), 7B2-PNU (500 ng/mL), or 7B2-pyPA (500 ng/mL), with or without CD4-IgG2 (500 ng/mL). Curves indicate the mean of triplicate samples (no SEM). (**B**) Area under the curves (AUC) in 293T or 92UG for the dgA or PNU immunoconjugates between the time of CIC addition and for a duration of 100 h. Unpaired *t*-test statistical analyses were performed using GraphPad Prism. ns: nonsignificant; * *p* < 0.05; *** *p* < 0.001; **** *p* < 0.0001. (**C**) Env expression on cell outgrowth following treatment with CIC. 92UG cells that remained at the completion of the time series shown in panel A were removed from plates and assayed for cell surface Env expression by indirect immunofluorescence and flow cytometry with anti-gp120 mAb VRC01.

In Figure 3A, we report the specificity of killing, demonstrate the CD4-mediated enhancement of the CIC effects, and replicate the outgrowth of cells following 7B2-dgA treatment, but not after 7B2-PNU. Specificity was indicated by the lack of effect of CICs on the growth of the Env-negative 293T cells and by the failure of a non-cytotoxic conjugate, 7B2-Py4pa (used for conjugating radionuclides), to affect 92UG cells. Statistical analyses performed on AUCs demonstrate that the CIC efficiencies on Env+ 92UG cells are statistically significant (*p* < 0.001) (Figure 3B). We have repeatedly shown that different forms of soluble CD4 can enhance the cytotoxic and antiviral effects of 7B2-dgA [4,12,42,43]. Here, we demonstrate a similar effect of CD4-IgG2 with 7B2-PNU. No enhancement of growth arrest by adding CD4-IgG2 to 7B2-dgA was observed because at the dosage used (50 ng/mL), a maximal effect was already obtained without CD4-IgG2.

In the untreated population of 92UG cells, approximately 5% of the cells were Env-negative (Figure 1A). We postulated that the outgrowth observed following treatment with 7B2-dgA (Figure 2A and Figure 3A) consisted of this Env-negative subpopulation, and that 7B2-PNU eliminated these cells through bystander effects. Figure 3C demonstrates cell surface Env expression on the cells shown in Figure 3A after they were continuously exposed to the CIC for 185 h. The cells growing out after 7B2-dgA treatment were largely Env-negative. Very few cells survived 7B2-PNU treatment, suggesting unspecific lysis of Env-negative cells by 7B2-PNU, which was not observed in 293T cells only (Figure 1B and Figure 3A). We postulate that the release of free PNU from cells ingesting the ADC thus kills nearby Env-negative cells. Following exposure to either CIC, there were low numbers of Env+ cells. Previous studies have examined the multiple mechanisms underlying resistance to IT killing that may explain this phenomenon [10,44,45,46].

**Figure 4:**
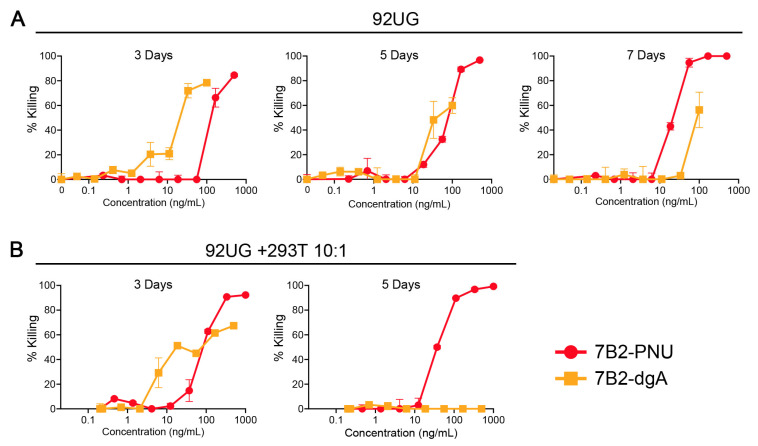
Dose–response of CICs over time on 92UG alone or mixed with 293T. Cells were cultured for the indicated time in the continual presence of CIC and CD4-IgG2 (500 ng/mL). Viability was quantified as MTS dye reduction over the final 3 h of culture. Panel (**A**) reports on 92UG cells alone, set up at 2 × 10^4^ cell/well for 3-day cultures, 10^4^ for 5 day, 5 × 10^3^ for 7-day cultures. Panel (**B**) reports on a mixture of Env+ and Env− cells set up at 2 × 10^4^ total cell/well for both 3- and 5-day cultures.

To test further for bystander-cell killing, we performed a mixing experiment in which 92UG was tested alone or mixed in a 10:1 ratio with parental 293T cells; killing was measured after exposure to varying concentrations of 7B2-dgA or 7B2-PNU (Figure 4). Figure 4A shows 92UG cells treated for different lengths of time. At 3 days, both CICs kill the vast majority of cells, with 7B2-dgA being ~10× more potent than 7B2-PNU. By 5 days, 7B2-PNU killed a greater proportion of cells than 7B2-dgA and was equally potent, and at 7 days 7B2-PNU killed all the cells and was 10× more potent. The results were even more pronounced when the Env-positive and Env-negative cells were mixed in a 10:1 ratio (Figure 4B). By 3 days, 7B2-PNU killed all the cells, whereas 7B2-dgA killed 50–60%. At 5 days, 7B2-dgA was completely ineffective due to the outgrowth of the Env-negative 293T. 7B2-PNU does not kill the Env-negative parental 293T cells at the concentrations tested (Figure 1B); instead, this bystander killing of 293T required the presence of Env-positive cells and was not observed if the initial population of Env-negative cells was >50% of the total population.

**Figure 5:**
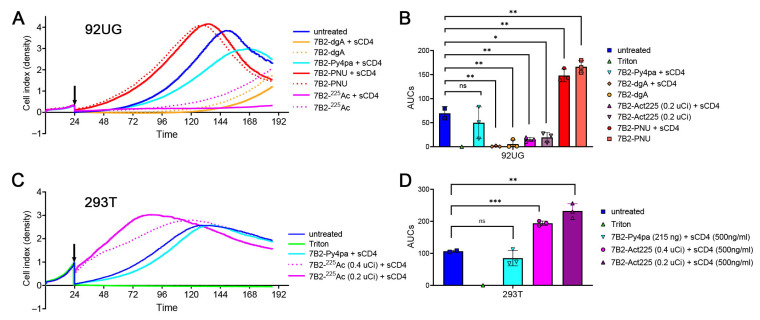
Effects of CICs, including RICs, with a short incubation period. Ten thousand cells (Env-expressing 92UG at top, Env-negative 293T at bottom) were plated and allowed to grow and adhere for 24 h. The indicated CIC was added to the monolayer (indicated with an arrow), incubated at 37 °C for 1 h, after which the CIC was removed, the plate was washed once with PBS, then fresh medium was added and the culture followed for 168 h. The following concentrations of CICs were tested: 7B2-dgA (125 ng/mL), 7B2-PNU (500 ng/mL), 7B2-Py4pa (215 ng/mL), 7B2-^225^Ac (0.2 µCi = 500 ng Ab and on 293T only 0.4 µCi) and CD4-IgG2 (500 ng/mL). The cell growth kinetic was measured every 15 min for 92UG (**A**) and 293T cells (**C**). Panels (**B**,**D**) show the area under the curves (AUC) in 92UG (**B**) or 293T (**D**) for the dgA-, PNU- or radio-immunoconjugates between the time of CIC addition and for a duration of 100 h. Unpaired *t*-test statistical analyses were performed using GraphPad Prism. ns: nonsignificant; * *p* < 0.05; ** *p* < 0.01; *** *p* < 0.001.

In Figure 5, the RIC 7B2-^225^Ac is added to our comparisons of the CICs. In the previous experiments (Figure 1, Figure 2, Figure 3 and Figure 4), the CICs were present for the duration of the incubation. We found that the RICs must be washed from the culture 1 h post-addition; otherwise pronounced non-specific toxicity is produced [33]. Therefore, in this experiment, CICs were washed from the cells after 1 h, and the cultures followed for the remaining time. CICs were tested on Env+ 92UG and Env-negative 293T cells. 7B2-dgA retained its full activity on 92UG (*p* < 0.01 compared to untreated) after this short incubation. 7B2-^225^Ac rapidly killed 92UG cells but not 293T. Killing by 7B2-^225^Ac was enhanced by the addition of sCD4. 7B2-Py4pa, the control conjugate without ^225^Ac, displayed cytotoxicity on neither 92UG nor 293T. Surprisingly, a significant enhancement of cell growth of 92UG cells was observed following a 1 h incubation with 7B2-PNU (*p* < 0.01). This growth enhancement was a reproducible phenomenon. We also noted a similar enhancement of growth following exposure of the 293T cells to both concentrations of 7B2-^225^Ac.

## 4. Discussion

A major goal of current anti-HIV therapy is to suppress infection sufficiently such that patients might remain free from anti-retroviral therapy (ART) for extended periods, i.e., obtain a durable remission. The reservoir of cells carrying a functional provirus that persists in the face of years of suppressive ART is a barrier to achieving this outcome. One approach towards eliminating this reservoir is to activate latent virus with latency-disrupting agents, leading to the immune-mediated killing of activated cells while protecting uninfected cells with ART, sometimes called “activate-and-purge” or “kick-and-kill” [22,47,48,49]. Initially, it was hoped that viral cytopathic effect or host immune responses would eliminate the reservoir cells, but while evidence of viral activation has been observed, the decline in the reservoir over time was not clinically meaningful. Recent studies utilizing monoclonal antibodies in combination with latency disruption have shown some success, presumably with the antibodies functioning through Fc-mediated cytotoxic effects [19,20,21]. Our approach uses CICs to further enhance the cytotoxic efficacy of anti-HIV antibodies.

Here, we compared the cytotoxic activity of three distinct forms of CICs, each targeted by the same mAb and tested on the same target cells: an IT, 7B2-dgA, an ADC, 7B2-PNU, and a RIC 7B2-^225^Ac. Using a technology that allows for detailed kinetic analyses, we observed differences in the rates of killing, the emergence of antigen-negative variants, bystander killing, and even the enhancement of cell growth. To our knowledge, this is the first direct comparison of the cytotoxic activity of an IT, ADC, and RIC. These in vitro findings may have clinical relevance, posing the following question: does the manner of cell killing play a role in the efficacy of CIC treatment? Although these studies directly address the use of CICs to eliminate HIV-infected cells, they also have implications for the design of CICs in treating cancer, other persistent viral infections, autoimmune disorders, and other illnesses.

The construction of the ADC and IT was similar, with each carrying 0–3 toxic molecules per antibody but differing in the linker used [30]. The RIC has a much lower ratio of toxic moiety to Ab, one ^225^Ac atom per 82 mAb molecules. In earlier work developing anti-HIV ITs we (a) demonstrated in vitro efficacy against a variety of cell types, (b) identified an optimal antibody for targeting CICs to HIV-infected cells [11,42,50], (c) showed that soluble CD4 and low-molecular-weight CD4 mimetics enhance the efficacy of anti-gp41 CICs [4,12,42,43], and (d) demonstrated the in vivo efficacy of 7B2-dgA in SCID mice carrying tumors of HIV-infected cells and in SHIV-infected viremic macaques with AIDS [4,51]. In these studies, 7B2-dgA was well tolerated, even in the sickest animals, but immunogenicity limited the duration of its effect in the macaques.

Ricin’s effect on cells has been studied for over a century [52,53,54,55]. Ricin A chain is an N-glycosidase that inactivates the large ribosomal RNA subunit, halting protein synthesis and inducing apoptosis. A ricin-based IT is internalized from the cell surface into endocytic vesicles, where reduction releases the free A chain. From there, it is transported in a retrograde fashion, via the Golgi and ER, into the cytoplasm, its site of action, where a single ricin A chain is capable of cleaving 3000 ribosomes per minute. 7B2-dgA’s rapidity of action, potency, and ability to deliver a lethal hit in a brief time most likely reflects the potent enzymatic activity of the ricin A chain.

PNU-159682 is a natural metabolite of the anthracycline chemotherapeutic, nemorubicin, produced in the liver through the microsomal CYP3A-mediated oxidation of nemorubicin [56]. PNU is reported to be >6000× more cytotoxic than doxorubicin [30,57]. ADCs constructed with PNU have been tested in animals and early clinical trials [57,58,59,60,61,62]. Anthracyclines are well recognized to function by intercalating into DNA and inhibiting topoisomerase II [63], and this is assumed to be the mechanism of PNU’s cytotoxicity as well [58]. However, due to its extreme toxicity, it has been suggested that PNU also affects other cellular functions, besides topoisomerase II inhibition [64]. Because PNU is bound to the antibody by a non-cleavable linker, proteases must first digest the antibody before free PNU is released [57]. This extra step of degradation may, in part, account for the delayed effect of 7B2-PNU on cell growth relative to 7B2-dgA. It also appears that 7B2-PNU requires a longer exposure to deliver the cytotoxic hit than does 7B2-dgA, suggesting that sustained intracellular concentrations of PNU are required to kill a cell. There were two unexpected observations regarding 7B2-PNU. The first was the observation of bystander cell killing, although this has been previously shown for ADCs [58,65]. In this case, the Env-negative bystander cells were either present in the original 92UG population or added 293T cells. To observe this bystander killing in vitro, the number of Env+ target cells must outnumber the Env-negative cells. We postulate that the bystander killing resulted from the release of highly toxic free PNU into the pericellular milieu as the targeted cell’s proteases digested it away from the antibody, followed by uptake of the free drug by adjacent Env-negative cells. Depending upon the clinical situation, the nonspecific killing of neighboring cells may be considered beneficial or an unwanted side effect. In HIV infection, where neighboring cells may also harbor transcriptionally silent provirus, bystander killing could be of benefit. In addition, it should be noted that due to the rarity of HIV-infected cells in ART-suppressed individuals, even in tissue sanctuaries, this constitutes a risk of targeting non-infected neighboring cells. This will require further investigations in vivo in HIV-infected and ART-suppressed animal models. The second unexpected observation was that low doses or short exposure to 7B2-PNU led to enhanced growth of Env+ cells (Figure 2B and Figure 5). This would not have been observed in conventional 3-day cytotoxic assays but was readily apparent in our kinetic analysis. Should this phenomenon be generalizable to other cell types and other toxic moieties, it could have implications for the clinical application of ADCs. Dosing and antibody formulation of the ADC may need to be adjusted to avoid the short exposure or suboptimal dosing that triggers DNA replication and cell growth.

^225^Ac is an α-emitting isotope with a half-life of ~10 days. It decays through a chain of short-lived daughters, collectively emitting four α-particles per decay and releasing a total energy of approximately 28 MeV. The emitted α-particles have high linear energy transfer (LET ~ 100 keV/µm), resulting in densely ionizing radiation that can induce double-stranded DNA breaks that initiate a chain of events leading to cell death. Unlike ITs and ADCs, the internalization of RICs is not required; only cell-surface binding is necessary for cell killing. The high-energy α particles can traverse the distance from the cell surface to the nucleus, as well as the nuclei of immediately adjoining cells [35]. Thus, RICs can kill both dividing and non-dividing cells, and extend their effects to adjoining cells. Our data (Figure 5) indicate that killing by 7B2-^225^Ac was specific, rapid, could be enhanced by sCD4, and did not result in outgrowth of variant cells, likely indicating bystander killing. The enhanced growth of Env-negative 293-T cells in the presence of 7B2-^225^Ac, where the RIC is mostly free in the medium and not attached to the cell surface, thus delivering a lower dose of radioactivity, is consistent with our hypothesis regarding enhanced growth following low-dose or short exposure to 7B2-PNU: the observed enhanced growth rate is a cellular response to sub-lethal injuries. RICs, including those incorporating ^225^Ac [66,67], have been investigated in clinical settings for cancer therapy and conditioning prior to stem cell transplantation, demonstrating encouraging results that highlight the potential of this radionuclide for clinical applications.

These CICs were evaluated on 92UG cells, a cell line stably transfected with the HIV Env. The advantage of this cell line relies on its stable expression of the HIV Env at the cell surface without cytopathic effects. Future studies aiming to test the clinical efficacy of anti-HIV CICs will be performed ex vivo in HIV-infected primary T cells or T cells from ART-suppressed people living with HIV (PLWH), as well as in vivo in HIV-infected and ART-suppressed humanized mice and/or non-human primate models. CICs could be ideal reagents for targeting the HIV reservoir since they can eliminate rare, widely dispersed cells such as those that constitute the persistent reservoir in patients infected with HIV. To date, most clinical trials using anti-HIV mAbs have employed broadly neutralizing antibodies (bNAbs), reasoning that their neutralization potential is key to their effectiveness. However, neutralizing antibodies frequently select for viral escape variants, which can rapidly diminish their effectiveness [68,69,70]. In contrast, the neutralization potential (and therefore the probability of viral escape) of mAbs used to construct ITs is not a determinant of their effectiveness [42]. It is plausible that the use of non-neutralizing antibodies, such as 7B2, to construct CICs will present a lower risk for antibody-driven viral escape, although this has yet to be tested in vivo. Additionally, combinations of neutralizing and/or non-neutralizing mAbs could be used to generate CICs targeting various Env epitopes. The observations made here warrant further investigation and comparison of these CICs in vivo. Future studies are planned that will focus on improvements in CIC design, testing comparative efficacy, using combinations of CICs, and optimizing the administration of CICs in HIV-infected humanized mice and in SHIV-infected macaques to assess the immunogenicity of each CIC and their antiviral function against HIV in these models.

## Data Availability

The original contributions presented in this study are included in the article. Further inquiries can be directed to the corresponding authors.

## Abbreviations

The following abbreviations are used in this manuscript:

293T	HEK 293T cells
7B2	anti-gp41 mAb
7B2-^225^Ac	anti-gp41 mAb 7B2 conjugated to the α-emitting isotope actinium-225
7B2-dgA	anti-gp41 mAb 7B2 conjugated to deglycosylated ricin A chain
7B2-PNU	anti-gp41 mAb 7B2 conjugated to anthracycline derivative PNU-159682
7B2-Py4Pa	a non-cytotoxic immunoconjugate made in preparation for use the construction of 7B2-^225^Ac
92UG	HEK-293T/92UG cells
ADC	antibody–drug conjugate
ADCC	antibody-dependent cellular cytotoxicity
ART	anti-retroviral therapy
AUC	area under the curve
bNAbs	broadly neutralizing antibodies
CICs	cytotoxic immunoconjugates
Env	envelope of HIV-1
HPLC	high-performance liquid chromatography
IT	immunotoxin
LET	linear energy transfer
mAb	monoclonal antibody
PLWH	People Living With HIV
PNU	PNU-159682
RIC	radioimmunoconjugate
RTCA	xCELLigence Real-Time Cell Analysis system
SEM	standard error of mean
